# Beyond Inflammation: Role of Pyroptosis Pathway Activation by Gram-Negative Bacteria and Their Outer Membrane Vesicles (OMVs) in the Interaction with the Host Cell

**DOI:** 10.3390/cells13211758

**Published:** 2024-10-23

**Authors:** Silvia Caterina Resta, Flora Guerra, Adelfia Talà, Cecilia Bucci, Pietro Alifano

**Affiliations:** 1 Department of Biological and Environmental Sciences and Technologies (DiSTeBA), University of Salento, Via Provinciale Lecce-Monteroni 165, 73100 Lecce, Italy; silviacaterina.resta@unisalento.it (S.C.R.); flora.guerra@unisalento.it (F.G.); adelfia.tala@unisalento.it (A.T.); 2 Department of Experimental Medicine (DiMeS), University of Salento, Via Provinciale Lecce-Monteroni 165, 73100 Lecce, Italy; cecilia.bucci@unisalento.it

**Keywords:** pyroptosis, host–pathogen interaction, outer membrane vesicles, virulence factors

## Abstract

Pyroptosis is a gasdermin-mediated pro-inflammatory programmed cell death that, during microbial infections, aims to restrict the spreading of bacteria. Nevertheless, excessive pyroptosis activation leads to inflammation levels that are detrimental to the host. Pathogen-associated molecular patterns (PAMPs) present in bacteria and outer membrane vesicles (OMVs) can trigger pyroptosis pathways in different cell types with different outcomes. Moreover, some pathogens have evolved virulence factors that directly interfere with pyroptosis pathways, like *Yersinia pestis* YopM and *Shigella flexneri* IpaH7.8. Other virulence factors, such as those of *Neisseria meningitidis*, *Neisseria gonorrhoeae*, *Salmonella enterica*, and *Helicobacter pylori* affect pyroptosis pathways indirectly with important differences between pathogenic and commensal species of the same family. These pathogens deserve special attention because of the increasing antimicrobial resistance of *S. flexneri* and *N. gonorrhoeae,* the high prevalence of *S. enterica* and *H. pylori*, and the life-threatening diseases caused by *N. meningitidis* and *Y. pestis*. While inflammation due to macrophage pyroptosis has been extensively addressed, the effects of activation of pyroptosis pathways on modulation of cell cytoskeleton and cell–cell junctions in epithelia and endothelia and on the bacterial crossing of epithelial and endothelial barriers have only been partly investigated. Another important point is the diverse consequences of pyroptosis pathways on calcium influx, like activation of calcium-dependent enzymes and mitochondria dysregulation. This review will discuss the pyroptotic pathways activated by Gram-negative bacteria and their OMVs, analyzing the differences between pathogens and commensal bacteria. Particular attention will also be paid to the experimental models adopted and the main results obtained in the different models. Finally, strategies adopted by pathogens to modulate these pathways will be discussed with a perspective on the use of pyroptosis inhibitors as adjuvants in the treatment of infections.

## 1. Introduction

Cell death can occur either accidentally as a consequence of excessive physical, mechanical or chemical injuries, or can be programmed through distinct cell suicide pathways, collectively termed regulated cell death (RCD). The latter includes (i) programmed cell death, necessary for development or tissue turnover that does not rely on any exogenous stimuli [1,2] and (ii) diverse forms of RCD that the cell activates depending on the stimuli received. Among these, pyroptosis is a programmed pro-inflammatory cell death mediated by members of the gasdermin family [3], first described in 1992 in macrophages infected by *Shigella flexneri* [4]. Pyroptosis plays an essential role in the innate immune response, inhibiting the replication of pathogens in the intracellular environment and activating immune cells to eradicate the infection [5,6].

To recognize pathogens, mammalian host cells use a broad array of pattern-recognition receptors (PRRs), which bind either conserved microbial molecules, collectively referred to as pathogen-associated molecular patterns (PAMPs), or host molecules known as damage-associated molecular patterns (DAMPs), which are released from stressed or damaged infected cells [7,8]. Among bacterial PAMPs, a vast array of structural molecules can be listed including lipopolysaccharide/lipooligosaccharide (LPS/LOS), peptidoglycan, lipoteichoic acid (LTA), lipoproteins and flagellin. DAMPs vary greatly depending on the type of cell and tissue damaged. DAMPs from intracellular compartments include molecules from: i. cytosol (heat-shock proteins, S100 proteins, cyclophilin A, F-actin, amyloid beta, ATP, uric acid); nucleus (histones, HMGB1, HMGN1, interleukin-1α, interleukin-33, SAP130, DNA, RNA); mitochondria (mtDNA, TFAM, formyl peptides, mROS); endoplasmic reticulum (calreticulin); granules (defensin, cathelicidins LL37, eosinophil-derived neurotoxin, granulysin); plasma membrane (syndecans, glypicans) [9]. DAMPs from the extracellular matrix include biglycan, decorin, versican, heparan sulfate, hyaluronan fragments, tenascin C, fibronectin and fibrinogen [9]. While pathogen recognition outside the host cell and in endosomal compartments is carried out by membrane-bound PRRs, such as Toll-like receptors (TLRs) and C-type lectin receptors, pathogen recognition in the cytoplasm is executed by nucleotide-binding oligomerization domain (NOD)-like receptors (NLRs) or Pyrin and HIN domain-containing (PYHIN) family proteins, RIG-1-like receptors (RLRs), and several cytosolic nucleic acid sensors, which bind PAMPs or DAMPs leading to the activation of pyroptosis [10,11].

Different pyroptosis cascades occur, culminating in the activation of gasdermins and cell death. The classical pathway relies on inflammasomes, cytosolic protein complexes consisting of (i) NLRs or PYHIN family proteins, (ii) the apoptosis-associated speck-like protein containing a caspase recruitment domain (CARD) (ASC) adapter protein and (iii) procaspase-1 [12,13]. The best-characterized inflammasome is NLRP3, and its activation is a tightly controlled process that involves two steps: inflammasome priming (signal 1) and inflammasome activation (signal 2). In inflammasome priming, nuclear activation of factor-κB (NF-κB) leads to the transcription of genes encoding the inflammasome components, pro-interleukin-1β (pro-IL-1β) and pro-interleukin-18 (pro-IL18) [14]. Furthermore, the large tumor suppressor kinases 1 and 2 (LATS1/2) are recruited to the microtubule-organizing centre (MTOC) and NLRP3 is palmitoylated at Cys958 (mouse Cys955) by the palmitoyl transferase zDHHC1 [15]. During the inflammasome activation, NLRP3 undergoes a second palmitoylation by zDHHC1 [15] or zDHHC7 [16] at Cys130 (mouse Cys126). These palmitoylations are required for the trafficking of NLRP3 between mitochondria, the trans-Golgi network (TGN), where it is assembled, and MTOC. Here, NLRP3 is phosphorylated by LATS1/2 at Ser265 (mouse Ser261) and interacts with NIMA-related kinase 7 (NEK7) [15]. In macrophages, palmitoylation at Cys898 has been found to occur on NLRP3 [17]. Inflammasome activation leads to self-cleavage of procaspase-1 which in turn cleaves and activates gasdermin-D (GSDMD), pro-IL-1β and pro-IL18. In the non-canonical pathway, caspase-11 (caspase-4 and -5 in humans) acts as an intracellular receptor for LPS/LOS of Gram-negative bacteria, directly binding lipid A portion of LPS leading to caspase-11 oligomerization and activation [18]. Active caspase-11 in turn cleaves GSDMD when a specific activation threshold is reached [19].

GSDMD has a C-terminal repressor domain (GSDMD-C) and an N-terminal pore-forming domain (GSDMD-N). Inflammatory caspases cleave the interdomain loop releasing GSDMD-N [19,20] which is palmitoylated at Cys192 by the palmitoyl acyltransferase zDHHC7 [21] or at Cys191 by zDHHC5 and zDHHC9 [22]. These modifications allow GSDMD-N translocation to the inner leaflet of the plasma membrane, where it interacts with specific phosphoinositides or cardiolipin [20,23]. GSDMD-N is subsequently depalmitoylated by the acyl protein thioesterase APT2, which promotes its oligomerization [21]. Thus, plasma membrane pores of 10–14 nm inner diameters are formed [20] through which pro-inflammatory cytokines, such as IL-1β and IL-18 are released [24]. IL-1β induces inflammation, vasodilatation and immune cell extravasation [25] while IL-18 stimulates the production of interferon-ɣ (IFN-ɣ) by Th1, natural killer and cytotoxic T cells, and promotes the local inflammatory response [26,27]. GSDMD cleavage and palmitoylation are independent events and cleavage-deficient GSDMD was found still palmitoylated upon inflammasome activation and was still able to cause pyroptosis, even if less efficiently than palmitoylated GSDMD-N [22].

**Figure 1:**
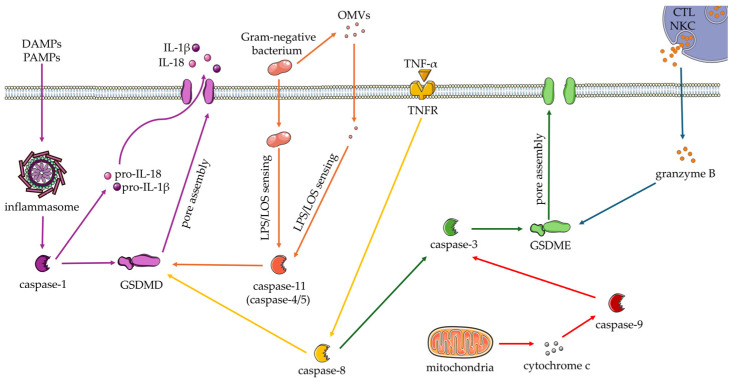
Pyroptosis pathway cascades. The activation of the different pyroptosis pathways involves distinct molecular players. In the canonical inflammasome pathway (purple path), Damage Associated Molecular Patterns (DAMPs) and/or Pathogen Associated Molecular Patterns (PAMPs) activate the inflammasome and thereby caspase-1. The latter in turn activates the pore-forming protein gasdermin-D (GSDMD) and the pro-interleukins pro-IL-1β and pro-IL-18 which are secreted through the GSDMD pores. In the non-canonical pathway (orange path), the lipopolysaccharide (LPS), exposed on the surface of cytosolic bacteria or Outer Membrane Vesicles (OMVs), is sensed by the caspase-11 which cleaves the GSDMD. Through the GSDMD pores, PAMPs and DAMPs are released and activate the inflammasome. Activation of the pyroptosis may also be inflammasome-independent. Indeed, the caspase-8, activated by the tumor necrosis factor-α (TNF-α), can cleave the GSDMD (yellow path) and the caspase-3. The caspase-3, in turn, cleaves the GSDME (green path). Caspase-3/GSDME interaction, moreover, can result from a switch from apoptosis to pyroptosis (red path). The GSDME can also be activated by granzyme B, released by cytotoxic T lymphocytes (CTL) and/or natural killer cells (NKC) (blue path). This Figure was created using Servier Medical Art, (https://smart.servier.com/), provided by Servier, licensed under a Creative Commons Attribution 3.0 unported license. Servier Medical Art is a service to medicine provided by Les Laboratoires Servier, Suresnes, Île-de-France, France (https://servier.com/).

In addiction to the canonical and non-canonical inflammasome, the caspase-3/gasdermin E (GSDME) and Caspase-8/GSDMD pathways have been described. In particular, tumor necrosis factor (TNF) triggers the activation of caspase-8 which cleaves GSDMD and activates caspase-3. Caspase-3 acts on GSDME causing its cleavage [28]. Moreover, the activation of the caspase-3/GSDME pathway may result from the switch from apoptosis to pyroptosis. Other members of the gasdermin family, such as the GSDMA, GSDMB, GSDMC and GSDMA3 (whose genes are present in the mouse but absent in the human genome), have pore-forming and pyroptotic activity [29,30]. Furthermore, members of the granzyme family can cleave gasdermins. In particular, granzyme B can directly cleave GSDME at the same site as caspase-3 [31] and granzyme A can directly cleave GSDMB [32]. The pyroptosis pathways described here are schematized in Figure 1.

## 2. Pyroptosis Pathways Activation by Gram-Negative Bacteria and Outcomes in the Host Cell

There is well-established evidence that pyroptosis is often useful in controlling infections. Indeed, the canonical pyroptosis pathway aims to limit the spread of intracellular bacteria by killing the host cell [5] and by trapping intracellular bacteria in the pore-induced intracellular traps (PITs) [33]. Nevertheless, excessive pyroptosis activation brings a level of inflammation that is detrimental to the host. It has been demonstrated to be involved in lethal septic shock induced by LPS [34,35]. Consistently, inhibition of pyroptosis via blocking GSDMD palmitoylation/depalmitoylation protected mice from LPS-induced septic shock [21]. The importance of this inflammatory pathway in host–pathogen interaction is inferred from the presence of some important pathogens, of virulence factors that interfere directly or indirectly with pyroptosis (Table 1). Moreover, exposure to commensals or pathogens belonging to the same family determines important differences in the activation and outcomes of pyroptosis pathways. Below we summarize how pyroptosis pathways are activated during the infection by Gram-negative bacteria and how several pathogenic bacteria exploit these pathways to accomplish their infectious cycle.

### 2.1. Neisseriaceae

*Neisseria* genus includes two important pathogens: *Neisseria gonorrhoeae* (the gonococcus) and Neisseria meningitidis (the meningococcus). *N. gonorrhoeae* is the causative agent of gonorrhea, which manifests as urethritis, cervicitis and/or extragenital infections (mainly pharynx, rectum and conjunctiva) [36]. The absence of a vaccine and the increasing resistance to available antibiotics together with non-mutational and non-hereditary forms of resistance make gonococcal infection an urgent threat [37,38,39,40,41]. *N. meningitidis* is a transitory colonizer of the human nasopharynx, which is occasionally responsible for the Invasive Meningococcal Disease (IMD) in some healthy carriers [42]. In fact, the meningococcus can breach the mucosal barrier, reaching the bloodstream and causing meningococcemia. Moreover, it can cross the blood–brain barrier (BBB) causing meningitidis [43]. IMD is a life-threatening disease and survivors have long-term sequelae [44,45]. Other members of the genus are the commensals *N. lactamica*, *N. mucosa*, *N. sicca*, *N. subflava*, *N. cinerea*, *N. elongata* and *N. flavescens*. These species colonize different human districts without causing disease and there is evidence that some of these species may antagonise the pathogenic *Neisseria* species [46,47,48,49,50].

Like few other bacteria, *N. gonorrhoeae* releases peptidoglycan fragments in quantities sufficient to cause inflammation and cytokine release. Tripeptide monomers are recognized by the Nucleotide Binding Oligomerization Domain 1 (NOD1) receptor, which induces NF-κB dependent production of TNF-α, IL-6, IL-8 and IL-1β in fallopian tube mucosa and macrophages [51,52,53]. The lytic transglycosidases LtgA and LtgD remodel cell wall and produce peptidoglycan monomers, which conversely suppress TNF-α and IL-1β by modulating the NOD2 and Toll-Like Receptor-2 (TLR2) signaling pathway in THP-1 macrophages [54]. *N. meningitidis* and non-pathogenic *Neisseria* spp. release fewer peptidoglycan fragments, and a smaller fraction of tripeptide monomers [55,56]. In THP-1 cells extrinsic apoptosis is inhibited by the gonococcus [57] and NLRP3-driven pyroptosis is induced [58]. Lack of caspase induction in these cells leads to activation of NLRP3 by cathepsin B [59]. This mechanism plays a role in the activation of the canonical pyroptosis pathway activation in THP-1 monocytes, since downregulation of cathepsin B downregulation reduces NLRP3 activation and IL-1β production by *N. gonorrhoeae* [58,59]. In the U937 cell line and human monocyte-derived macrophages, the gonococcus inhibits both intrinsic and extrinsic apoptosis [58], and in human macrophages both canonical and non-canonical pathways are activated [60,61]. In monocyte-derived macrophages (MDMs) *N. gonorrhoeae* induces caspase-1 activation, but exogenous ATP is required for IL-1β secretion [62]. Pyroptosis in MDMs was subsequently associated with intracellular bacteria and it was prevented by the caspase-1 inhibitor Z-WEHD-FMK or the caspase-4 inhibitor Z-YVAD-FMK but not by the caspase-3 inhibitor Z-DEVD-FMK. Furthermore, a mutant strain with hypo-acylated (penta-acylated instead of hexa-acylated) lipid A portion of LOS, known to induce decreased cytokine production in epithelial cells [63], failed to prevent caspase-1 or caspase-4 activation in MDMs [60]. Pyroptotic cell death in macrophages did not affect the viability of gonococci and it was demonstrated that pyroptosis induction requires viable bacteria [59]. Caspase-1 is required for the processing of pro-IL-1β but was dispensable for cell death which instead was found to be dependent on NLRP3 and cathepsin B [59]. In THP-1 cells secretion of IL-1β is also induced by *N. cinerea* and *N. flavescens* but these commensal species are weak inductors compared to *N. gonorrhoeae* [58].

While the gonococcus induces cell death in macrophages, monocytes and B cells, in neutrophils and epithelial cells the gonococcus inhibits this process. In epithelial cells, gonococcal-dependent NF-κB activation provides protection against Receptor-interacting serine/threonine-protein kinase 1 (RIPK1)-dependent necroptosis and inhibits apoptosis [64]. However, with low bacterial loads, the gonococcus triggers the apoptosis by translocation of PorB into the inner mitochondrial membrane and by activation of Rac-1 by Opa proteins, which leads to activation of the proapoptotic proteins Bax and Bak [65,66]. All *Neisseria* spp. express PorB porin, but the PorB amino acid sequence differs between the species. Both PorB from *N. gonorrhoeae* and *N. meningitidis* translocate PorB to mitochondria, with opposite results: gonococcus PorB induces apoptosis, whereas meningococcal PorB inhibits this process [67,68]. Moreover, the translocation of PorB seems to be pathogen-specific since *N. mucosa* PorB does not colocalize with mitochondria [69]. Gonococcal PorB has also been shown to induce a transient increase in calcium levels in cells, which activates calpain [70], an enzyme with roles in both apoptosis and pyroptosis [71,72,73,74]. Calpain has been reported to mediate cell disruption during pyroptosis through vimentin filament cleavage and loss of intermediate filaments. Calpain-dependent cell rupture was dispensable for IL-1β release but required for the release of mitochondria and bacteria [71]. There is also evidence that *N. gonorrhoeae* induces an upregulation of NLRP3 protein in the endometrial cell line hEECs, which is dramatically inhibited in TLR2- or TLR4-silenced cells [75] while gonococci-infected human endocervical epithelial cells (End/E6E7) undergo RIPK1-dependent necroptosis [60]. Asymptomatic or subclinical infections by *N. gonorrhoeae* are much more common in females (50–80%) than in males (1–3%) [76,77,78,79]. It has been proposed that progesterone plays a role in this clinical manifestation. Higher serum progesterone levels correlate with asymptomatic gonorrhea and with low IL-1β levels in cervical secretion [80], and in a murine model progesterone was able to reduce the levels of IL-1β, IL-6 and TNF-α levels in vaginal secretion, neutrophil infiltration and the number of polymorphonuclear neutrophils. Progesterone also decreases NLRP3 protein and IL-1β mRNA levels, and represses caspase-1 activity in genital tissue and THP-1 cell line [81].

In purified human primary monocytes challenged with *N. meningitidis*, apoptosis is induced when bacterial concentration is low, whereas pyroptosis is activated when meningococci are abundant. In contrast, the infections by *Escherichia coli* and *Klebsiella pneumonieae* induce pyroptosis regardless of the bacterial load [82]. This process is associated with the loss of intracellular ATP in *E. coli* and *K. pneumonieae* infections. The meningococcus, conversely, inhibits the glycolysis and the oxidative phosphorylation in infected cells, but upregulates the hypoxia-inducible factor-1α (HIF-1α) transcription factor, preserving the intracellular ATP levels [82]. Importantly, Webster and colleagues [82] observed pro-inflammatory cytokines released by monocytes infected with *N. meningitidis* but not by cells infected with *E. coli* or *K. pneumonieae*. *N. lactamica* also preserves the intracellular host cell ATP levels, but cytokines release is different in cells infected with *N. lactamica* compared to cells infected with *N. meningitidis*, with less release of IL-1β and more release of the antiinflammatory IL-10 [82]. On the other hand, elevated levels of IL-10 have been found in the plasma of patients with fulminant meningococcal sepsis patients 8–24 h after the first symptoms [83]. IL-10 has been shown to prevent NLRP3 and RIPK2 upregulation in *N. meningitidis*-infected human monocytes as well as IL-1β release, while IL-10-treated monocytes upregulate caspase-5 when infected with the meningococcus. The inflammasome AIM2, which oligomerizes upon recognition of bacterial DNA, was upregulated in infected monocytes, an effect enhanced by IL-10 treatment [84]. In co-culture experiments, Tezera and colleagues demonstrated that *N. lactamica* abolished meningococcal-induced inflammation through inhibition of NF-κB in the epithelial Detroit 565 cell line but not in meningeal cells. Modulation of NF-κB activity modulation is achieved by the upregulation of Peroxisome proliferator-activated receptor-ɣ (PPAR-ɣ), which controls the availability of NF-κB in the nucleus [85].

Neisserial LOS, which activates the non-canonical inflammasome, is an important inductor of pyroptosis. It is phosphorylated in the lipid A portion and the degree of this phosphorylation correlates with the inflammatory potential and severity of IMD [86]. *N. meningitidis* lipid A is pyrophosphorylated and phosphoethanolaminylated while *N. gonorrhoeae* lipid A has a reduced phosphorylation and lower pro-inflammatory activity [87]. Among commensal Neisseria species, only *N. lactamica* and *N. elongata* possess a functional LptA enzyme to transfer phosphoethanolamine to lipid A [88]. Meningococcal LOS phosphorylation also impacts the expression of miR-146a, a microRNA that negatively regulates NF-κB and inflammation [89]. The most highly inflammatory LOS is also the greatest inducer of miR-146a [90]. Meningococcal strains with the most highly inflammatory and phosphorylated lipid A were more restricted to the central nervous system of patients and had reduced capacity to cause septicemia. Conversely, strains with less phosphorylated LOS are more capable of inducing systemic infections [86]. Guanylate binding proteins (GBPs) are interferon-inducible GTPases involved in innate immunity response. GBPs are required for non-canonical inflammasome recognition of intracellular bacteria promoting LPS release and presentation to caspase-11/4 [91,92]. Recently, it has been reported that GBP1 and GBP3, through their N-terminal domain, selectively kill *Francisella novicida* and *N. meningitidis* but not other bacterial or mammalian cells. GBPs-mediated disruption of bacteria exposes the intracellular content for inflammasome sensing. GBP1 was found to be active against both wild-type meningococcus and the lpxA-defective mutant, which has no LOS [93]. Another factor that plays a role in Neisseria-induced pyroptosis is hemagglutinin/hemolysin-related protein A (HrpA), the secreted portion of the two-partner HrpA/HrpB secretion system [94]. HrpA acts as a manganese-dependent cell lysin and mediates the bacterial escape from the internalization vacuole [95,96]. Through this mechanism, the meningococcus reaches the cytosol and is recognized by inflammasomes. In addition, HrpA binds to the motor protein dynein, enabling the bacterium to move along the microtubules. hrpA- and hrpB-defective meningococci were strongly impaired in the activation of both canonical and non-canonical pyroptosis pathways in vitro [94] and in vivo in a murine model of meningitis [97]. In HeLa cells, NSC34 motor neuron-like cells and hBMEC brain endothelial cells the caspase-3/GSDME pathway and the caspase-11 (caspase-4) were activated by meningococcus infection, with subsequent activation of caspase-1 [94]. In contrast, a prevalence of GSDMD-mediated pyroptosis was observed in the brains of meningococcus-infected BALBc mice. Furthermore, in infection with an hrpB-defective mutant, together with a reduction in the activation of pyroptosis pathways, an increase in animal survival was observed compared to infection with wild-type meningococci [97].

### 2.2. Enterobacteriaceae

The family of Enterobacteriaceae comprises ubiquitous Gram-negative bacteria with 33 genera to date. These include human pathogens, the most studied of which are *Salmonella* and *Shigella* spp. [98], with *S. flexneri* being the first microorganism discovered to induce pyroptosis [4].

*S. flexneri* is the causative agent of bacillary dysentery, an acute intestinal infection that occurs following ingestion of contaminated food and water. Bacillary dysentery is characterized by intestinal inflammation, abdominal pain, cramps, diarrhea and fever, and accounts for more than 250 million cases worldwide each year [99]. *S. flexneri* crosses the intestinal epithelial barrier through M cells and is then endocytosed by resident macrophages and dendritic cells. After entering the cell, *S. flexneri* lyses the vacuole and reaches the cell cytosol [100]. Several virulence factors of *S. flexneri* have been shown to activate the pyroptosis or interfere with it. *Shigella* possesses a functional type III secretion system (T3SS), and recognition of the TS33 inner rod protein MxiI by Naip2 activates the NLRC4 inflammasome and caspase-1, leading to secretion of IL-1β and IL-18 and pyroptosis [5,101,102,103]. *Shigella* infection triggers the apoptotic signal of the Tumor protein p53 (TP53) in epithelial cells, but the bacterium can prevent the induction of apoptosis by cleaving the calpain inhibitor calpastatin through the VirA factor. Sustained activation of calpain instead leads to necrotic cell death. However, *S. flexneri* lacking the T3SS factor OspC3 triggers caspase-4 dependent-pyroptosis in HaCaT keratinocyte and HT29 epithelial-derived cell line [101,104]. OspC3, in particular, catalyzes a post-translational modification, arginine ADP riboxanation, of caspase-4 Arg314 and caspase-11 Arg310. These modifications block the autocatalytic cleavage of caspases and the recognition and activation of the GSDMD. Mice infected with *S. flexneri* Δ*ospC3* survived the infection, in contrast, wild-type or OspC3-complemented strains caused animal death [105]. *S. flexneri* factor IpaH7.8, a member of the E3 Ubiquitin ligase-like family, targets GSDMB and GSDMD for degradation in infected cells [106,107]. GSDMB is not activated by caspases, but granzyme A released by cytotoxic T lymphocytes and natural killer cells activates it directly [32]. GSDMB accumulation is observed following *Shigella* infection when the 26S proteasome is blocked by MG132 treatment [107]. IpaH7.8 mimics host E3 ubiquitin ligase, binding and ubiquitinating GSDMB and GSDMD on multiple Lys residues. Interestingly, IpaH7.8 can target human GSDMD but not mouse GSDMD [106]. Another member of the IpaH protein family, IpaH9.8, targets guanylate-binding proteins (GBPs) for degradation [92,108,109].

*Salmonella enterica* serovar Typhimurium (herein referred to as *S. typhimurium*) is a pathogen that can cause acute and chronic infections. Clinical manifestations vary from asymptomatic carriage, gastroenteritis and systemic disease [110]. It possesses T3SSs encoded by *Salmonella* Pathogenicity Islands (SPI) SPI-1 and SPI-2 through which it secretes virulence factors into the host cell [111,112]. Another important virulence factor for this pathogen is the flagellum. Its subunit, flagellin, consists of conserved D0 and D1 domains (N-terminal and C-terminal) and hypervariable D2 and D3 domains (central region) [113]. D1 domain binds to TLR5 while D0 domain binds to Naip5/6 triggering the assembly and activation of the NLRC4 inflammasome [113,114]. When *S. typhimurium* lacks the flagellin genes *fliB* and *fljC*, group 3 innate lymphoid cells (ILC3s) from C57BL/6-infected mice failed to produce IL-22 [115], a cytokine proved to enhance *Salmonella* mucosal dissemination [116]. Conversely, this cytokine was produced when using wild-type *Salmonella* and also mutated strains for T3SS SPI-1 and/or SP-2. Pyroptotic cell death was detected in ILC3s of *S. typhimurium*-infected mice, regardless of IL-22 or *Salmonella* flagellin production. This cell death was dependent on caspase-1 and GSDMD, since disulfiram (GSDMD inhibitor) and Ac-YVAD-cmk (caspase-1 inhibitor) prevented it. Although flagellin was essential for IL-22 production in ILC3s and dispensable for cell death, mice lacking caspase-1 had more ILC3 cells with less cell death, more IL22-producing ILC3 cells and higher mortality compared to wild-type-infected mice. Therefore, control of ILC3s pyroptosis appears to play a role in *S. typhimurium* infection [115]. Consistent with these findings, pyroptosis has been proven to be beneficial to the host in the early stages of *Salmonella* infection preventing bacterial dissemination [117,118,119]. Caspase-1 deficient C57BL/6 mice die from *Salmonella* oral administration [120]. Caspase-8 and NLRC4 inflammasomes also play a role in limiting *Salmonella* infection [118]. On the other hand, in systemic infection, pyroptosis has been shown to be harmful to mice. Caspase-1 or GSDMD deficiency in the intraperitoneal infection model increased mice survival with attenuated secretion of IL-1β, IL-6 and TNFα [121]. Besides flagella, the T3SS needle and inner rod also activate Niap/NLRC4 in mice and human macrophages [5,114,122,123,124,125,126]. In primary human monocytes, the NLRP3 inflammasome was found to be activated following exposure to *S. typhimurium* or LPS. This activation was accompanied by the secretion of IL-1β and IL-1α but without pyroptosis. Treatment with MCC950 blocked IL-1β and IL-1α secretion in *S. typhimurium*-infected cells but only IL-1β secretion in cells exposed to LPS alone [127]. However, NLRC4 and NLRP3 inflammasomes are dispensable for pyroptosis activation in human intestinal Caco-2 cells, as is NLRC4 in enteroids exposed to *S. typhimurium* infection. In human intestinal cells infected with *Salmonella*, CRISPR/Cas9 system, used to disrupt *CASP4*, revealed that caspase-4 is essential to activate the inflammasome [128]. This activation leads to IL-18 secretion from Caco-2 and T84 cells and is SPI-1 dependent. This difference could be explained by the different levels of expression of inflammasome components by macrophages and epithelial cells, the latter having lower mRNA levels of caspase-1, NLRP3, NLCR4 and Niap [128]. *Salmonella* plasmid virulence C protein (SpvC) is a T3SS effector that plays a role in bacterial dissemination in mice [129,130]. It reduces pyroptosis in the cecum of C57BL/6 mice and mouse J774A.1 macrophages [129,130,131,132]. *spvC* mutants trigger the activation of the NLRP3 and NLRC4 inflammasomes in mouse cecum, unlike wild-type *S. typhimurium* [129]. SpvC shares 63% amino acid identity with *S. flexneri* OspF and has the same phosphothreonine lyase activity on MAPK [131,132]. This enzymatic activity is essential for SpvC to suppress pyroptosis [129]. SPI-1 effectors include SopE, an activator of Rho GTPase, which has been linked to pyroptosis pathways. It induces caspase-1 activation and IL-1β release in RAW264.7 murine macrophages as well as inflammation in infected mice [133]. While SopE plays a critical role in this process, canonical inflammasome activation in these cells is not dependent on flagella [134]. Moreover, SopE contributes to the egress of *Salmonella* from the *Salmonella*-containing vacuole (SVC) in macrophages [135]. Pyroptosis induction by SopE was found to be dependent on its activity on Rac1 and Cdc42 [101,133]. In intestinal epithelial cells, another critical T3SS effector, SopF, was found. Most C57BL/6 mice infected with *Salmonella* ∆*sopF* survived infection with increased levels of IL-1β release and inflammation compared to mice infected with wild-type *Salmonella*. Infections of Caco-2 cells and normal human colonic epithelial NCM460 cells revealed that SopF inhibits GSDMD-mediated and, especially, the GSDME-mediated pyroptosis through the caspase-3/GSDME pathway, by inactivating the caspase-8. In contrast, SopF promotes the necroptosis [136]. *Salmonella* regulates the length of LPS O-antigen by FepE. Low expression of FepE, as in *S. typhimurium*, is associated with an increased capacity to trigger pyroptosis in macrophages [137]. *S. paratyphi*, which express FepE [138], or *S. typhimurium* mutants overexpressing FepE are weaker inductors of pyroptosis compared to wild-type *S. typhimurium* strain [137].

Polycystic ovary syndrome (PCOS) correlates with gut microbiome dysbiosis and increased abundance of *Enterobacteriaceae* in the gut of patients [139]. In the mouse model of PCOS, an increased abundance of Gram-negative bacteria (*Desulfovibrio* and *Burkholderia*), an increase in circulating LPS and reduction in the abundance of *Akkermansia* were found compared to the control group. Leakage of LPS into the circulation induces GSDMD-dependent pyroptosis in macrophages, and this process may damage receptor complexes on the plasma membrane, disturbing the epithelial integrity. Treatment of PCOS mice (in which PCOS was induced by dehydroepiandrosterone) with disulfiram or metformin reduced pyroptosis in macrophages and increased gut levels of *Akkermansia*, which helps reinforce the intestinal barrier and reduce LPS leakage [140].

### 2.3. Yersiniaceae

The genus *Yersinia* includes three human pathogens: *Y. pestis*, *Y. pseudotuberculosis* and *Y. enterocolitica*. *Y. pestis* is the causative agent of the plague, which is present in stable foci in America and Africa. The disease has five main forms: bubonic, septicemic, pneumonic, meningeal and pharyngeal plague [141,142]. Infections with *Y. pseudotuberculosis* and *Y. enterocolitica* are widespread throughout the world, both causing gastroenteritis, while *Y. pseudotuberculosis* can also cause mesenteric lymphadenitis [143,144]. These bacteria are characterized by a virulence plasmid coding for a T3SS through which they inject into the host cell *Yersinia* outer proteins (Yops) [145]. Among these, many Yops are dedicated to perturbing host cell death pathways. Replication of *Y. pestis* and *Y. pseudotuberculosis* in the host is initially silent with low inflammation and is subsequently accompanied by cytokines production and tissue necrosis [146,147,148,149,150]. This disease course correlates with apoptotic cell death of naïve macrophages and, later in the infection, with pyrototic cell death of activated macrophages [24]. YopJ in *Y. pestis* and *Y. pseudotuberculosis* (named YopP in *Y. enterocolitica*) leads to caspase-8-dependent macrophage cell death. It inhibits the MAP kinase Transforming Growth Factor Beta-Activated kinase 1 (TAK1) by acetylation. When TAK1 is inhibited, TNF activation causes RIP1 to dissociate from complex I to form complex IIa with FADD and procaspase-8 [151]. If caspase activity is blocked, RIP1 dissociates from complex IIa to form complex IIb with RIP3, which induces MLKL-dependent necroptosis [152]. Naïve macrophage apoptosis requires YopJ [24]. Inactivation of caspase-8 and RIP3 or inactivation of RIP1 protects bone marrow-derived macrophages (BMDMs) from *Y. pseudotuberculosis*-induced cell death but leads to the death of infected animals [153]. The strong IL-1 response during *Yersinia* infection, which requires activation of the NLRP3 inflammasome, is crucial for the animal death [154]. *Yersinia*-induced caspase-8 activation leads to pyroptosis in macrophages with both GSDMD and GSDME activation. Cleavage of GSDMD and GSDME is abolished in *Rip3^-/-^ Casp8^-/-^* macrophages, whereas *Casp3/7^-/-^* macrophages show inhibition of GSDME activation alone. However, human macrophages behave differently. Indeed, when human peripheral blood mononuclear cell (PBMC)-derived macrophages, the U937 cell line and monocyte-derived macrophages are infected with *Y. pseudotuberculosis*, YopJ-dependent cell death is not observed. The level of IL-1β secretion in these cells is comparable to that of macrophages exposed to LPS. Pretreatment of PBMC-derived macrophages with the TAK1 inhibitor 5z7 induces pyroptotic cell death with GSDME activation but the absence of GSDMD activity, suggesting that in humans TAK1 inhibition can be overcome [155]. Caspase-8 and RIP1 activation was also demonstrated in *Y. enterocolitica*-infected mouse bone marrow-derived dendritic cells [156]. YopK is a virulence factor that has been shown to inhibit NLRP3 inflammasome activation in vivo without affecting YopJ-induced cell death. *Y. pseudotuberculosis* expressing YopK but lacking YopJ does not induce T3SS-dependent inflammasome activation and cell death in mouse BMDMs [157]. However, priming of macrophages with inflammatory stimuli is sufficient to induce pyroptosis in macrophages infected with *Yersinia* YopJ-deficient bacteria [24]. In cells lacking NLRP3, ASC or NLRC4, caspase-1 is activated and IL-1β and IL-18 are still secreted upon *Yersinia* infection, suggesting that other inflammasomes are involved [157,158]. Considering the interconnection between apoptosis, pyroptosis and necroptosis, the concept of PANoptosis has recently emerged. In this cell death, members of apoptosis, pyroptosis and necroptosis are simultaneously engaged in the PANoptosome complex and the pathway cannot be blocked by the terminal effectors of the individual pathways [159,160,161]. In *Yersinia*-infected macrophages, RIP1 is essential in the induction of apoptosis and pyroptosis. Its ablation inhibits apoptosis and pyroptosis but enhances necroptosis. RIP1 mediates the PANoptosome assembly. Compared with wild-type BMDMs, *Gsdmd^-/-^* BMDMs, *Casp3^-/-^* BMDMs, *Casp7^-/-^* BMDMs, *Mlkl^-/-^* BMDMs *Rip3^-/-^* BMDMs and *Casp1/11^-/-^* BMDMs did not show impaired cell death when infected with *Y. pseudotuberculosis*. *Casp1/11^-/-^* BMDMs, however, had impaired IL-18 secretion [162]. Conversely, cell death was found to be reduced in *Rip3^-/-^ Casp8^-/-^* BMDMs and especially in *Rip3^-/-^ Casp8^-/-^ Casp1/11^-/-^* BMDMs. The impact of RIP1 in *Yersinia* infection was investigated in fetal liver-derived macrophages (FLDMs) since RIP1 ablation is lethal to mice and BMDMs cannot be generated. In *Rip1^-/-^* mice, FLDMs exhibited spontaneous MLKL activation and *Yersinia* infection led to reduced activation of caspase-1, GSDMD, caspase-3, caspase-7 and caspase-8 compared to wild-type-infected FLDMs [162]. Recently, it has been found that *Y. pseudotuberculosis* infection increases glycolysis and reduces intracellular glucose levels in BMDMs and this leads to the glucose- and energy-responsive activation of AMPK, which in turn phosphorylates RIP1 during the caspase-8-mediated pyroptosis [163]. The IFN-ɣ inducible Z-DNA binding protein, ZBP1, has been shown to play a role in the assembly of the RIPK1-TRIF-caspase-8 complex in response to *Yersinia* infection [164].

Other Yop proteins are involved in pyroptosis pathways. YopE and YopT are Rho-modifying enzymes, YopE is a GTPase-activating protein while YopT is a protease. RhoA modifications are sensed by Pyrin inflammasome which activates caspase-1, leading to IL-1β secretion and cell death [165]. YopE activates Pyrin by triggering its dephosphorylation at Ser205 [166], which appears to be a conserved mechanism since the *Clostridium difficile* RhoA-inactivating enzyme TcdB acts in the same way on Pyrin [165]. In BMDMs YopE and YopT can induce pyroptosis only in the absence of YopM, a virulence factor which inhibits pyrin inflammasome [166]. This inflammasome is expressed mainly in immune cells, such as macrophages, cytokine-activated monocytes and granulocytes but also in serosal and synovial fibroblasts [167]. YopT similarly dephosphorylates Pyrin although more slowly than YopE [166]. Thus, the appropriate mixture of Yop proteins delivered to the host cell governs its fate.

### 2.4. Helicobacteriaceae

The *Helicobacteriaceae* family includes two genera: *Wolinella* and *Helicobacter*. The latter genus comprises 35 species among which *Helicobacter pylori* is the most studied [168]. *H. pylori* is found in almost 50% of the world’s population, it targets the stomach and it is the main cause of different gastrointestinal diseases [169]. These include gastric and duodenal ulcers, mucosa-associated tissue lymphoma and gastric adenocarcinoma [170]. NLRP3 is the inflammasome most widely reported to be activated by *H. pylori*, both in vitro [171,172,173] and in vivo [174,175,176]. NLRP3 inflammasome was found to be activated in the stomach of *H. pylori*-infected mice with the Muc1 mucin playing a protective role [174]. In human peripheral blood mononuclear cells (PBMCs), *H. pylori* induces NLRP3 inflammasome activation and IL-1β release [177]. The cytotoxin-associated gene pathogenicity island (cagPAI) is critical in the ability of *H. pylori* to induce pyroptosis. This genomic island encodes a type IV secretion system (T4SS) through which the bacterium injects, among others, one of its major virulence factors, CagA [178]. A component of the T4SS involved in CagA translocation and adherence to host cells is CagL [179]. Another important virulence factor is VacA, a pore-forming toxin secreted by the type V secretory system (T5SS). In *H. pylori*-infected dendritic cells, TLR2 dependent-NLRP3 inflammasome activation and IL-1β secretion are reduced in the absence of cagPAI or CagL but not in the absence of CagA or VacA [180]. In contrast, in the human gastric mucosal epithelial cell line GES-1 and in the human gastric epithelial adenocarcinoma cell line AGS CagA is sufficient to activate NLRP3 inflammasome via reactive oxygen species (ROS) production. Inhibition of ROS production by N-acetyl-l-cysteine blocks NLRP3 inflammasome and pyroptosis [181]. Similarly, in THP-1 monocytes, *H. pylori*-induced pyroptosis depends on NLRP3 activation via ROS production [171]. Central to *H. pylori* infection is the production of urease, which catalyzes the production of ammonia and carbonic acid from urea, neutralizing gastric acidity. Urease protein is composed of six UreA subunits and six UreB subunits with two coordinated nickel ions into each UreB [182]. UreB has been found to play a role in pyroptosis. *ureB*-deficient mutants induce IL-1β transcription in mouse bone marrow-derived dendritic cells (BMDCs) but fail to activate the NLRP3 inflammasome and secrete IL-1β. Similar to cagPAI [180], UreB-mediated activation of NLRP3 is TLR2 dependent [183]. NLRP3 and GSDMD expression is increased in gastric mucosal samples from *H. pylori*-infected subjects compared to the control group. Rabeprazole, a proton pump inhibitor used in the treatment of gastric ulcers, effectively reduced GSDMD cleavage and secretion of IL-1β and IL-18 in BGC823 cells [184]. Pyroptosis in the gastric mucosa of *H. pylori*-infected subjects has been associated with the transition from chronic gastritis to gastric cancer, which has been found to be promoted by CagA [185]. The anthraquinone derivative emodin and the BCF-01 strain *Weizmannia coagulans*, isolated from a healthy subject, showed protective effects against *H. pylori* through the downregulation of bacterial virulence factors and the inhibition of pyroptosis [186,187]. In particular, the expression of CagA, VacA and CagL was reduced in emodin-treated *H. pylori* compared to untreated bacteria. Furthermore, IL-1β and IL-18 secretion was shown to be reduced in *H. pylori*-infected AGS cells when treated with emodin, as well as the activation of caspase-1 and GSDMD, and the translocation of VacA [186]. *H. pylori* decreases the expression of tight junction proteins in C57BL/6 mice and the GES-1 cell line. Mice or cells pretreated with *W. coagulans* BCF-01 showed normal tight junction expression levels when infected with *H. pylori*. Moreover, mice and RAW264.7 macrophages exposed to the same treatments showed a strong reduction in GSDMD and caspase-1 activation, along with reduced IL-1β and IL-18 secretion compared to untreated cells infected with *H. pylori*-infected ones [187]. Importantly, the authors of this study found that BCF-01 was more effective in reducing the activation of the pyroptosis pathways than the triple antibiotic treatment (amoxicillin, clarithromycin and omeprazole) used in *H. pylori* infection [187].

**Table 1:** Virulence factors that are involved in the activation or regulation of pyroptosis pathways.

Bacterium	Virulence Factor	Role in Pyroptosis	Reference
*Neisseria gonorrhoeae*	PG monomers	NOD1-dependent IL-1β release	[53,54,56]
	PorB	Calpain activation	[70]
*Neisseria meningitidis*	LOS	Non-canonical pathway activation, miR-146a expression induction	[89,90]
	HrpA	Vacuole escape and consequent exposure to inflammasome sensing	[94,97]
*Shigella flexneri*	MxiI	NLRC4-dependent pyroptosis	[103]
	OspC3	Inactivates caspase-4 and caspase-11 by ADP riboxanation	[104,105]
	IpaH7.8	Targets GSDMD and GSDMB for degradation	[106,107]
	IpaH9.8	Targets GBPs for degradation	[109]
*Salmonella enterica*	Flagellum	NLRC4 activation	[114,115]
	T3SS needle	NLRC4 activation	[114,123,124,125,126]
	SpvC	Reduces NLRP3 and NLRC4 activation via phosphothreonine lyase activity on MAPK	[129,130,131,132]
	SopE	Induces caspase-1 activation dependent on its activity on Rac1 and Cdc42	[133,134,135]
	SopF	Inhibits GSDMD and caspase-3/GSDME pyroptosis	[136]
*Yersinia pestis* *Yersinia pseudotuberculosis* *Yersinia enterocolitica*	YopJ, YopP *	Activation of caspase-8/GSDMD and caspase-3/GSDME pyroptosis in mice	[154]
	YopK, YopQ *	Inhibits NLRP3 inflammasome	[157]
	YopE and YopT	Activate Pyrin inflammasome through its dephosphorylation	[165,166]
	YopM	Inhibits Pyrin inflammasome	[154]
*Helicobacter pylori*	CagL	Impairs TLR2-dependent NLRP3 activation in dendritic cells	[180]
	CagA	Activates NLRP3 inflammasome via ROS production in epithelial cells	[181,185]
	UreB	Impairs TLR2-dependent NLRP3 activation	[183]

* Yop proteins names in *Yersinia enterocolitica.*

## 3. Epithelial and Endothelial Barriers Crossing: Can Pyroptosis Pathways Help Bacteria?

Epithelial and endothelial barriers are the first line of defense against pathogens. On the other hand, several pathogens have developed strategies to overcome or disrupt these barriers to gain access and invade host tissues. Recent pieces of evidence reveal a crucial role of inflammation caused by pyroptosis pathways in the integrity of these barriers. In ulcerative colitis, gut microbiota dysbiosis can cause pyroptosis. Levels of pore-forming GSDME-N have been correlated to tissue inflammation and intestinal barrier integrity in patients. Low expression of zonula occludens-1 (ZO-1), E-cadherin, and occludin (OCLN) was found in the mucosa of ulcerative colitis patients together with activation of caspase-3 and GSDME [188]. Recently, 4 octyl itaconate (4-OI) has been demonstrated to inhibit the caspase-3/GSDME pathway [189]. The use of butyrate-decorated liposomes carrying 4-OI (4-OI/Blipo) in NCM460 cells, treated with TNF-α to trigger the activation of caspase-3, reduced cell death, GSDME-N levels and re-established the levels of tight junction proteins. Pyroptosis disruption of tight junction increases the epithelial permeability and aids bacteria to cross the epithelial barrier. Consistently, dextran sulfate sodium (DSS)-induced colitis mice have reduced GSDME-N levels in colonic epithelium and increased expression of occludin and E-cadherin after treatment with 4-OI/Blipo [188]. In primary human gingival epithelial cells (HGECs), sodium butyrate activates GSDME-mediated pyroptosis. At the same time, it was observed a reduction in mRNA levels of gap junction genes [Connexin 26 (*Cx26*), *Cx43*], adherence junction gene Cadherin-1 (*CDH1*), tight junction genes [Junction Adhesion Molecule-1 (*JAM-1*), Claudin-1 (*CLDN1*) and *CLDN4*] and desmosome genes [Desmoglein-1 (*DSG1*) and Desmocollin-2 (*DSC2*)]. Immunostaining reveals that E-cadherin and claudin-1 were disturbed by sodium butyrate [190]. Anaerobic Gram-negative bacteria produce butyrate. The inflammation caused by these bacteria is associated with periodontitis and epithelial disruption is the first step of the pathology [190,191]. Pyroptosis activation in epithelia does not always favour bacterial crossing, but can also contrast it. Although excessive activation of Niap/NLRC4 pyroptosis in intestinal epithelial cells (IECs) contributes to the disruption of the gastrointestinal barrier, this pathway protects mice against *Salmonella* infection [118,192]. *S. typhimurium*-infected IECs are extruded from the intestinal epithelium. On the contrary, in the absence of *Niap* genes, the extrusion is reduced, and it is accompanied by *Salmonella* invasion of the epithelium in mice [117,118]. Extrusion was found to be independent of IL-18, IL-1α or IL-18 [117]. Besides pyroptosis, the apoptosis pathway can also induce the extrusion of infected cells [193,194] with some differences. Contraction of the epithelium aids the closure of epithelial gaps during the extrusion of infected cells in small intestine organoids. This process was found to be Niap/NLRC4-dependent and ion flux through GSDMD pores is a necessary signal for contraction [192,195]. C57BL/6 mice exposed to LPS have reduced expression of ZO-1, claudin-1 and occludin proteins and activation of GSDMD-mediated pyroptosis in the ileum and colon compared to the control group. Du and colleagues found that pretreatment of mice with the carotenoid fucoxanthin ameliorates LPS toxicity with reduced pyroptosis and recovery of tight junction protein expression [196].

**Figure 2:**
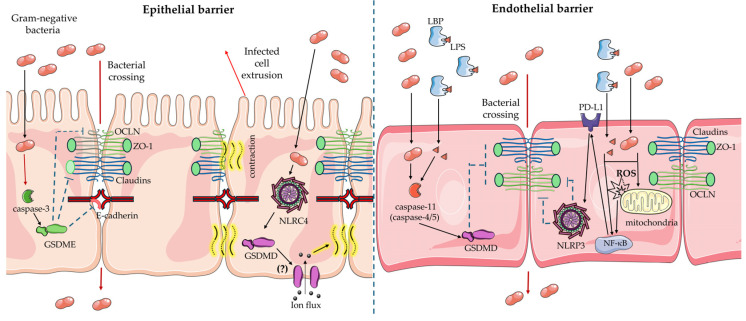
Pyroptosis activation effects on the epithelial and endothelial barrier. Gram-negative bacteria activate the caspase-3/GSDME pathway in epithelial cells. This activation can disrupt tight and adherence junctions by interfering with occluding (OCLN), zonula occludens-1 (ZO-1) and E-cadherin, thus facilitating bacteria to cross the epithelium. Activation of NLRC4 inflammasome in epithelial cells leads to gasdermin-D (GSDMD) activation and pore formation. It is not known if GSDMD pores are formed on the apical or basolateral side or both the functional domains of the epithelial cells. GSDMD-mediated ion flux leads to tissue contraction to extrude the infected cell. In endothelial pyroptosis, caspase-11 activation by LPS interferes with tight junctions. Moreover, reactive oxygen species (ROS) from mitochondria can induce Programmed Death Ligand-1 (PD-L1) expression and NLRP3 activation. This leads to a reduced expression of ZO-1 and OCLN. This Figure was created using Servier Medical Art (https://smart.servier.com/), provided by Servier, licensed under a Creative Commons Attribution 3.0 unported license. Servier Medical Art is a service to medicine provided by Les Laboratoires Servier, Suresnes, Île-de-France, France (https://servier.com/).

LPS also disrupts the BBB [197,198]. Evan blue extravasation measure and observation of ultrastructural changes revealed that LPS impairs BBB in C56BL/6 mice with abnormal tight junction appearance but not in *Casp11^-/-^* or *Gsdmd^-/-^* deficient mice. Disruption of BBB was found to be dependent on activation of non-canonical inflammasome and not on TLR4 cytokines induction, although the LBP-CD14-TLR4 axis is necessary for LPS internalization. Moreover, GSDMD activation in the absence of LPS is sufficient to induce BBB disruption [199]. Pyroptosis in endothelium was recently related to the expression of Programmed Death Ligand 1 (PD-L1), which normally inhibits excessive T-cell activation [200]. LPS treatment in human lung microvascular endothelial cells (HMVECs) reduces the occludin and ZO-1 expression only in the presence of PD-L1. Moreover, overexpression of PD-L1 in these cells is sufficient to decrease tight junction protein expression and to increase NLRP3 expression and activation of caspase-1. Pyroptotic death induced by PD-L1 overexpression was found to be mitochondrial ROS production-dependent and increases when PD-L1 overexpression is accompanied by LPS treatment [201]. Figure 2 schematizes how pyroptosis pathways activated by bacteria can interfere with epithelial and endothelial barriers.

## 4. Outer Membrane Vesicles (OMVs) as Carriers of Pathogen-Associated Molecular Patterns (PAMPs) That Trigger Pyroptosis Pathways

Outer membrane vesicles (OMVs) are released from the outer membrane of Gram-negative bacteria. OMVs are implicated in cell–cell communication, quorum sensing, stress responses and pathogenesis [202]. OMVs contain various pathogen-associated molecular patterns (PAMPs), including LPS, which induces the TLR4 signaling pathway and TLR4-dependent endocytosis [203]. LPS can also be internalized into the cell where it activates the non-canonical inflammasome. Indeed, circulating LPS can bind to high mobility group box 1 (HMGB1) which promotes its internalization through the Receptor for advanced glycation end products (RAGE) [204], expressed by various cell types, such as endothelial cells, smooth muscle cells, mesangial cells, mononuclear phagocytes and certain neurons [205]. In contrast, OMVs are internalized through clathrin-mediated endocytosis [206]. Recently, galectin-3 has been found to be implicated in internalization of LPS and subsequent activation of non-canonical pyroptosis [207]. Galectin-3 is an amphoteric polysaccharide-binding protein that can repeatedly cycle in and out of the cell. It is secreted by macrophages and binds, among other molecules, LPS [208]. Galectin-3 induces internalization of circulating LPS through RAGE. Galectin-3 also appears to mediate OMV internalization, but in a RAGE-independent manner since galectin-3 inhibition, but not RAGE silencing, was able to alleviate OMV-triggered pyroptosis [207]. Once inside the cell, LPS or OMVs require GBPs to activate non-canonical pyroptosis. Indeed, mouse BMDMs lacking GBPs or caspase-11 were strongly impaired in caspase-1 activation and secretion of IL-1β [209]. In *E. coli* infections, caspase-11 was predominantly activated by OMVs [206] and subsequent cell death required the expression of GBP2, while GBP5 was dispensable. In addition, mice lacking GBP2 exhibited higher survival rates when treated with OMVs compared to wild-type mice [209].

*S. typhimurium* and *Pseudomonas aeruginosa* OMVs trigger activation of NLRC4 inflammasome and secretion of IL-1β in mouse BMDMs. Inflammasome activation is dependent on flagella as OMVs derived from flagellin-deficient mutants of *Salmonella* could not activate NLRC4 and weakly activated the NLRP3 inflammasome [210]. In contrast, OMVs from the non-flagellated *E. coli* BL21 strain were found to strongly activate the NLRP3 inflammasome and GSDMD in BMDMs. In C57BL/6 mice, however, IL-1β secretion was dependent on both NLRC4 and NLRP3 inflammasomes [210]. Of note, GBP2 ablation was sufficient to prevent pyroptosis triggered by *E. coli* OMVs but not that triggered by *Salmonella* OMVs [210]. In agreement with this finding, Deo and colleagues found that *E. coli* OMVs triggered caspase-11-dependent cell death of BMDMs [211]. In contrast, caspase-11 was dispensable for cell death induced by *N. gonorrhoeae* OMVs. The authors of this study [211] found that the lipid A in *Neisseria* OMVs is modified by phosphoethanolamine which masks phosphates important for caspase-11 recognition [211]. Instead, *N. gonorrhoeae* OMVs were found to activate BCL-2 antagonist killer (BAK)-dependent intrinsic apoptosis and, in turn, NLRP3 inflammasome and IL-1 secretion [211]. Meningococci release abundant OMVs, which are important in the proinflammatory response in the early stages of IMD [86]. *N. meningitidis* OMVs trigger IL-1β secretion in human neutrophils [212]. In addition, both *N. gonorrhoeae* and *N. meningitidis* OMVs contain a large amount of PorB, which has been shown to be sufficient to induce apoptosis with *N. gonorrhoeae* OMVs [67].

**Figure 3:**
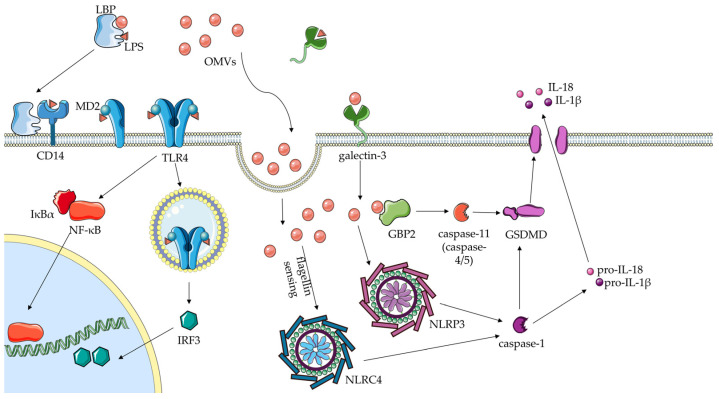
Outer membrane vesicles (OMVs) internalization and activated pyroptosis pathways. OMVs are internalized into the host cell by clathrin-dependent endocytosis or by a novel mechanism of internalization involving galectin-3. Once inside the cell, the OMVs are targeted by the guanylate binding protein 2 (GBP2) which is needed for the caspase-11 activation. Moreover, OMVs can activate NLRC4 inflammasome through flagellin and/or NLRP3 inflammasome. In addition, the lipopolysaccharide (LPS), exposed on the surface of OMVs, can be extracted by the LPS binding protein (LBP) and transferred to CD14 which releases the LPS to the TLR4-MD-2 complex. Activation of the TLR4 leads to nuclear factor-kappa B (NF-κB) activation or inflammatory endocytosis, which activates interferon regulatory factor 3 (IRF3). This Figure was created using Servier Medical Art (https://smart.servier.com/), provided by Servier, licensed under a Creative Commons Attribution 3.0 unported license. Servier Medical Art is a service to medicine provided by Les Laboratoires Servier, Suresnes, Île-de-France, France (https://servier.com/).

*E. coli* OMVs have also been found to trigger disseminated intravascular coagulation (DIC) in mice [213,214] via non-canonical inflammasome activation [214]. Calcium influx through GSDMD pores activates the phospholipid scramblase transmembrane protein 16S (TMEM16F). This enzyme mediates the externalization of phosphatidylserine, which binds and activates the tissue factor (TF) to initiate the coagulation cascade [215]. Mice intraperitoneally injected with *E. coli* OMVs exhibited features of systemic coagulation activation with increased serum levels of thrombin-antithrombin (TAT), plasminogen activator inhibitor type-1 (PAI-1) and D-dimer, and decreased fibrinogen plasma levels. Deletion of caspase-11 or GSDMD in mice significantly attenuated the activation of the coagulation cascade triggered by OMVs [214]. GSDMD and caspase-1 were activated in THP-1 macrophages exposed to OMVs from the gastrointestinal resident bacterium *Desulfovibrio fairfieldensis*, resulting in the secretion of IL-1β along with several other cytokines. Moreover, Caco-2 cells exposed to *D. fairfieldensis* OMVs showed reduced ZO-1 and occludin expression [216]. *Porphyromonas gingivalis* does not induce activation of pyroptotic pathways, but its OMVs elicit pyroptotic cell death in murine and human macrophages with caspase-1 activation and IL-1β secretion. This response was found to be dependent on heat-labile components of OMVs, as heat inactivation of OMVs prevented pyroptosis [217]. Figure 3 illustrates how OMVs can be internalized by the host cell and the pyroptotic pathways that OMVs can trigger.

## 5. Pyroptosis Inhibitors for the Treatment of Infectious Diseases

Inhibition of several mediators of pyroptosis is attracting increasing interest in the treatment of inflammatory diseases, and the safety and efficacy of some pyroptosis inhibitors are being evaluated in clinical trials. The pan-caspase irreversible inhibitor Emricasan (IDN-6556) was tested in non-alcoholic steatohepatitis because of its preferential distribution in the liver (ClinicalTrial.gov [https://clinicaltrials.gov/, accessed on 13 September 2024] ID: NCT02077374; NCT02686762 and NCT03205345) but was discontinued in Phase I clinical trial [218,219,220]. Emricasan was also tested for *Staphylococcus aureus* skin infections in mice and demonstrated effectiveness in reducing the size of the lesions and the bacterial load [221]. Pralnacasan (VX-740) and belnacasan (VX-765) inhibit caspase-1 activation. Pralnacasan was tested for rheumatoid arthritis but showed high liver toxicity in mice at high doses [218,222]. Instead, belnacasan entered clinical trials for the treatment of epilepsy (ClinicalTrial.gov [https://clinicaltrials.gov/, accessed on 13 September 2024] ID: NCT01501383 and NCT01048255) and psoriasis (ClinicalTrial.gov [https://clinicaltrials.gov/, accessed on 13 September 2024] ID: NCT00205465) [197] but further studies were stopped due to liver toxicity in prolonged treatment [218]. Nevertheless, belnacasan could be of interest for the treatment of sepsis. It was able to mitigate the depletion of immature transitional B cells and resting memory B cells in peripheral blood mononuclear cells (PBMCs) from septic shock patients [223]. Additionally, belnacasan alleviated the BBB disruption and cognitive dysfunction in a mouse model of sepsis [224]. Another caspase-1 inhibitor, AC-YVAD-CMK, efficiently alleviated renal injury, with reduced accumulation of neutrophils and macrophages in the cecal ligation and puncture mouse sepsis model [225]. MCC950 is a potent inhibitor of NLRP3 activation that can also inactivate already activated inflammasomes. Treatment with MCC950 was found to alleviate colonic inflammation in Winnie mice, with reduced infiltration of neutrophils and reduced secretion of IL-1β and IL-18 [226]. The Winnie mouse is a model of spontaneous chronic colitis in which bacterial dysbiosis has been shown to play a role in colonic inflammation [227,228]. A sodium-glucose cotransporter 2 (SGLT2) inhibitor exerted cardiovascular protection through the modulation of the NLRP3 inflammasome [229]. SGLT2 inhibitors are currently undergoing a Phase 4 clinical trial for the treatment of PCOS (ClinicalTrial.gov [https://clinicaltrials.gov/, accessed on 13 September 2024] ID: NCT05966792), another condition in which the gut microbiota plays a critical role [230,231]. Another promising strategy is to block the assembly or activation of GSDMD, although only a few inhibitors are currently available. One of the most studied GSDMD inhibitors is disulfiram, a drug currently in use for alcohol addiction, which inhibits the aldehyde dehydrogenase, and is subsequently found to inhibit GSDMD pore formation and therefore the pyroptosis without affecting the necroptosis [232]. It has entered different clinical trials for various conditions, such as cocaine abuse (ClinicalTrial.gov [https://clinicaltrials.gov/, accessed on 14 September 2024] ID: NCT00580827, NCT00218608, NCT00395850, NCT00000278, NCT00094289, NCT00913484, NCT00729300, NCT00142844 and NCT00149630), HIV infection (ClinicalTrial.gov ID: NCT00878306, NCT01944371, NCT01286259 and NCT00002065), breast cancer (ClinicalTrial.gov [https://clinicaltrials.gov/, accessed on 14 September 2024] ID: NCT03323346), SARS-CoV-2 infection (ClinicalTrial.gov [https://clinicaltrials.gov/, accessed on 14 September 2024] ID: NCT04485130 and NCT04594343), prostate cancer (ClinicalTrial.gov [https://clinicaltrials.gov/, accessed on 14 September 2024] ID: NCT01118741) and glioblastoma (ClinicalTrial.gov [https://clinicaltrials.gov/, accessed on 14 September 2024] ID: NCT02678975, NCT01907165, NCT03034135 and NCT02715609). Disulfiram could be of interest for sepsis. Treatment with a dosage within the approved clinical range increased the survival of mice with LPS-induced sepsis [232]. Similarly, another GSDMD inhibitor, necrosulfonamide, improved survival in a mouse model of sepsis [233]. Recently, Granzyme A inhibition through 4-octyl itaconate was shown to inhibit GSDMB-mediated pyroptosis and alleviate inflammation in a mouse model of colitis [234]. Pyroptosis inhibitors have not currently entered clinical practice for infectious diseases, and further studies in animal models are needed to achieve this result. Nevertheless, the crucial role of pyroptosis in the outcome of some bacterial infections and recent evidence in mouse models suggest that these inhibitors could represent a valuable weapon for the treatment of inflammation associated with many infectious diseases.

## 6. Conclusions

Cells activate several processes in response to PAMPs and DAMPs. Among these processes, pyroptosis plays a crucial role in bacterial infections. It plays a role in contrasting bacterial replication and bacterial clearance. On the other hand, excessive pyroptosis activation is harmful to the host and helps bacteria in invasion by crossing the epithelial and/or endothelial barrier. Important pathogens that cause diseases characterized by strong inflammation activate diverse pyroptosis pathways with different outcomes in different tissues. Shedding light on the mechanisms underlying the activation of pyroptosis pathways and the strategies adopted by bacteria to interfere with them is essential for the development of new drugs useful in the treatment of infectious diseases.

## Data Availability

Not applicable.
